# Lymphocyte-albumin-neutrophil ratio predicting short-term and long-term mortality risk in elderly patients with delirium: an analysis based on the MIMIC-IV database

**DOI:** 10.3389/fneur.2026.1740291

**Published:** 2026-04-23

**Authors:** Shu Yang, Shuo Zhang, Lianzheng Ma, Guoqing Li, Guowei Zhu, Yifei Cao, Minmin Zhu

**Affiliations:** 1 Wuxi School of Medicine, Jiangnan University, Wuxi, China; 2 Department of Anesthesiology and Pain Medicine, Wuxi No.2 People's Hospital (Jiangnan University Medical Center), Wuxi, China; 3 Tianjin Key Laboratory of Ionic-Molecular Function of Cardiovascular Disease, Department of Cardiology, Tianjin Institute of Cardiology, The Second Hospital of Tianjin Medical University, Tianjin, China

**Keywords:** elderly patients with delirium, LANR, long-term mortality, MIMIC-IV database, short-term mortality

## Abstract

**Background:**

Inflammatory response and nutritional status are closely related to the prognosis of critically ill patients, especially in elderly patients with delirium, where the situation is more complex. This study aims to explore the relationship between the Lymphocyte-Albumin-Neutrophil Ratio (LANR) and short- and long-term mortality risks in elderly delirium patients, providing valuable insights for clinical management.

**Methods:**

This study utilized the MIMIC-IV ICU public database to identify elderly delirium patients based on ICD-9 and ICD-10 codes. First, a qualitative approach was employed using Restricted Cubic Spline (RCS) analysis to visually illustrate the potential relationship between LANR and 30-day and 365-day mortality rates. Second, from a quantitative perspective, a Cox proportional hazards model was used for multivariable regression analysis to assess the independent effect of LANR on short- and long-term mortality risks after adjusting for other confounding factors. Additionally, to further explore heterogeneity, subgroup analyses were conducted, and forest plots were generated to examine potential significant interactions.

**Results:**

A total of 1,804 elderly delirium patients were extracted from the MIMIC-IV database, with a mean age of 77.6 ± 7.9 years and an ICU length of stay of 3.3 days [IQR 1.8–6.7 days]. Restricted cubic spline analysis revealed a linear relationship between LANR and 30-day mortality risk reduction in elderly delirium patients (*P* for non-linearity>0.05). Although LANR showed a non-linear relationship with 365-day mortality risk, the risk decreased progressively as LANR levels increased. Cox multivariable regression analysis, after adjusting for covariates, indicated that higher LANR levels (T2, 0.21 < LANR≤0.47) were associated with an 26% reduction in short-term mortality risk and an 18% reduction in long-term mortality risk [HR_30-day_ = 0.74, 95% CI: 0.60–0.91, *p* = 0.004; HR_365-day_ = 0.82, 95% CI: 0.70–0.96, *p* = 0.013]. Extremely high LANR levels (T3, LANR>0.47) were associated with a 45% reduction in short-term mortality risk and a 40% reduction in long-term mortality risk [HR_30-day_ = 0.55, 95% CI: 0.44–0.69, *p* < 0.001; HR_365-day_ = 0.60, 95% CI: 0.51–0.71, *p* < 0.001]. Subgroup analysis further identified a significant interaction between LANR and vasopressor use in relation to 365-day mortality (*p* = 0.018 < 0.05).

**Conclusion:**

The inflammatory marker LANR is closely associated with increased short- and long-term ICU mortality risk in elderly delirium patients, highlighting its potential as a prognostic tool in clinical practice.

## 1 Introduction

Delirium is an acute and fluctuating neuropsychiatric syndrome marked by attention deficits, cognitive dysfunction, and acute alterations in the level of consciousness, often accompanied by emotional or behavioral disturbances (1). The incidence varies across clinical settings: approximately 25% among elderly inpatients in general hospitals (2), with higher rates observed in cardiovascular intensive care units (CICU), showing a significant correlation with age (*p* < 0.05) (3). In emergency departments, the prevalence among elderly patients ranges from 8 to 17%, while in long-term care facilities, such as nursing homes, it can reach up to 79% (1, 4). The prognosis of delirium in older adults is influenced by multiple factors, including age, comorbidities, clinical context, subtype of delirium, nutritional status, and length of hospital stay. For example, advanced age (>85 years), coexisting cognitive impairment or depression significantly elevates the risk of delirium and correlates closely with poor outcomes (5). In CICU, delirium is associated with a significantly increased mortality rate during hospitalization and follow-up (OR = 3.2) (3). Elderly patients undergoing emergency surgery for hip fractures who develop delirium exhibit higher mortality rates and poorer functional recovery (6). Malnutrition, indicated by low Geriatric Nutritional Risk Index (GNRI) or Prognostic Nutritional Index (PNI) scores, is also strongly linked to the development of delirium and its adverse outcomes (7). Adverse clinical outcomes of delirium in older adults include elevated mortality (a 2- to 3-fold increase in in-hospital mortality and a 3-fold increase in 6-month mortality for ICU patients) (8), long-term cognitive decline [with some patients progressing to dementia (9)], loss of autonomy, and increased risk of institutionalization (10). Furthermore, delirium predisposes patients to complications such as nosocomial infections, pressure ulcers, falls, delayed postoperative recovery, and impaired functional rehabilitation (11). Despite the implementation of multidisciplinary interventions and systematic screening for high-risk individuals, no specific pharmacological treatments are currently available, leaving non-pharmacological interventions as the primary approach (12). Thus, further investigation into prognostic indicators and intervention strategies for delirium in older adults is crucial for improving patient outcomes.

Inflammatory mechanisms play a pivotal role in the development and prognosis of delirium in elderly patients, and various inflammatory blood markers have been validated as having significant predictive value. Notably, the neutrophil-to-lymphocyte ratio (NLR), serving as a systemic inflammatory marker, is strongly associated with both the onset and prognosis of delirium. Elevated NLR values signify heightened systemic inflammation and immune imbalance, potentially increasing delirium risk or worsening its prognosis by exacerbating intracerebral inflammation and oxidative stress (13). Furthermore, preoperative elevation in NLR has been significantly linked to an increased risk of postoperative delirium in elderly non-cardiac surgery patients, particularly when NLR exceeds 6.3, where the risk of delirium approximately doubles (14). Emerging markers such as the systemic immune-inflammation index (SII) and the prognostic nutritional index (PNI) demonstrate promising potential for assessing chronic inflammation and nutritional status, contributing valuable insights into the prognosis of delirium in elderly populations (15). Albumin levels, which reflect nutritional status and inflammatory regulation, also exhibit substantial prognostic significance. Low serum albumin levels indicate malnutrition and chronic inflammation, likely impairing antioxidant and anti-inflammatory defenses and thereby aggravating the pathophysiological processes underlying delirium (16, 17). The integration of inflammatory markers that comprehensively reflect inflammation, nutrition, and immune status may offer more precise tools for evaluating delirium prognosis in elderly patients. Nevertheless, further research is warranted to validate the specific application value of these emerging markers in elderly delirium and their correlation with clinical outcomes.

While multiple inflammation- or nutrition-related biomarkers—such as the systemic immune-inflammation index (SII) and prognostic nutritional index (PNI)—have been proposed for risk stratification in critically ill and elderly populations, each captures only discrete aspects of the underlying pathophysiology. SII primarily quantifies systemic inflammatory burden, whereas PNI focuses on assessing nutritional status and immune function; notably, neither has been adequately validated for use in elderly patients with delirium. Given that delirium in older adults arises from complex interactions between inflammatory responses, immune dysregulation, and malnutrition, a composite biomarker integrating these dimensions may offer greater pathophysiological relevance and enhanced prognostic discriminatory power. The lymphocyte–albumin–neutrophil ratio (LANR) combines three key parameters: lymphocyte count (reflecting immune function), neutrophil count (inflammatory activity), and serum albumin level (nutritional status), thereby potentially providing a more holistic assessment of physiological vulnerability in elderly patients. We hypothesized that LANR would be independently associated with both short-term and long-term mortality risk in elderly patients with delirium and could serve as a clinically actionable prognostic indicator in the intensive care unit (ICU) setting. Accordingly, this study aimed to systematically evaluate the association between LANR and mortality outcomes using data from the MIMIC-IV database. By conducting a retrospective analysis of clinical data from elderly patients with delirium, this study will further examine the association between LANR and both short-term and long-term patient outcomes.

## 2 Materials and methods

### 2.1 Data source

This retrospective study utilized publicly accessible data from the MIMIC-IV database. The author SY was granted full access to the MIMIC-IV database and took responsibility for extracting the relevant data (Credential ID: 62274870). This study strictly followed the guidelines outlined in the Strengthening the Reporting of Observational Studies in Epidemiology (STROBE) statement.

### 2.2 Research population

We included patients in the study who experienced their first ICU admission and were concurrently diagnosed with delirium. The diagnosis of delirium was based on the International Classification of Diseases, Ninth Revision (ICD-9) and Tenth Revision (ICD-10). Specifically, delirium was identified using the following ICD-9 codes: 29281, 2,930, 2,931, 2,939, 34,831, 34,982, 78,009, 78,097, and the following ICD-10 codes: F05, G92, G9341, R410, R4182 (18).

**Figure 1:**
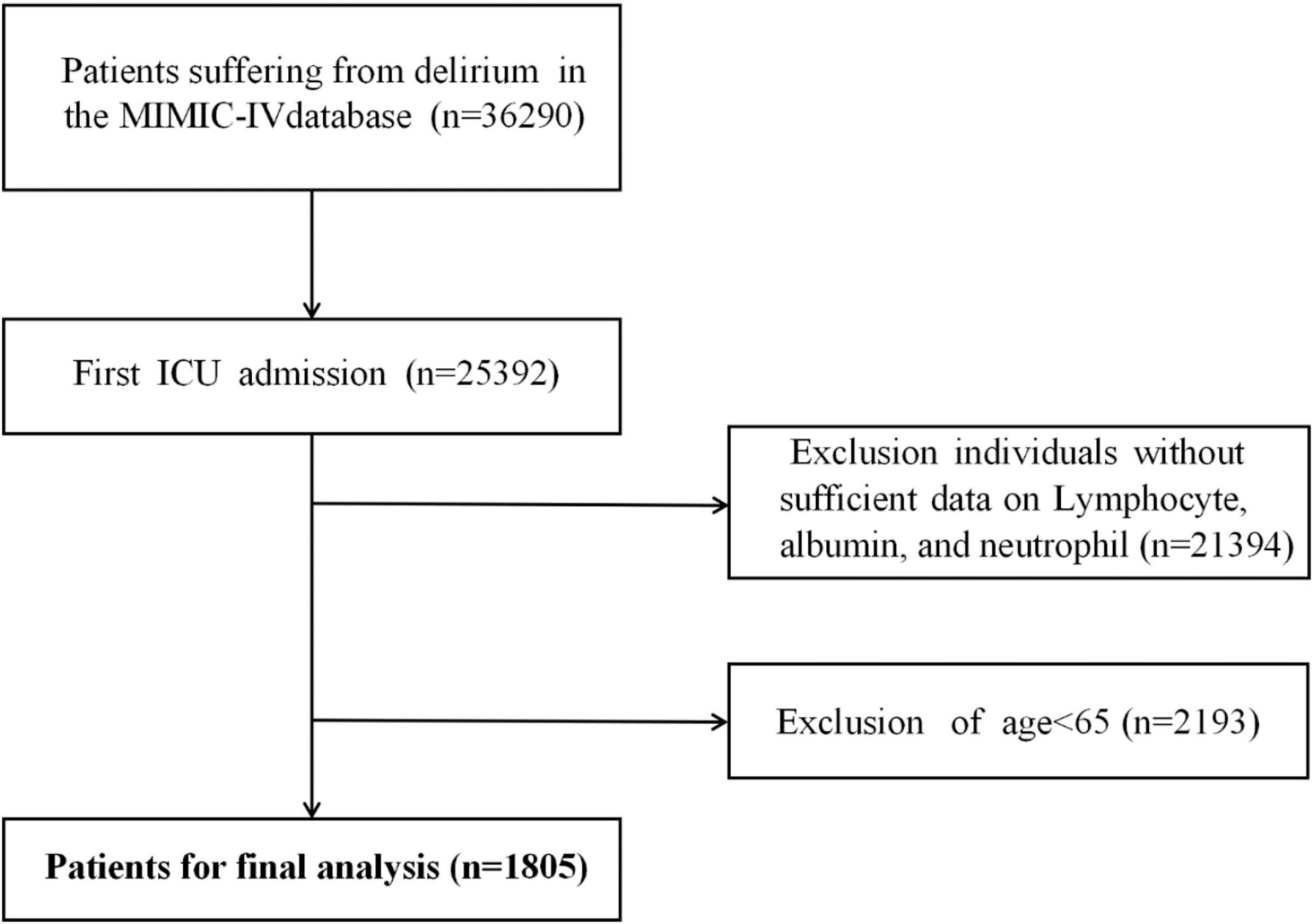
Flow chart of study population inclusion and exclusion.

The inclusion criteria were as follows: (1) age ≥ 65 years; (2) first ICU admission. Patients were excluded if they had missing data for lymphocytes, albumin, or neutrophils. After applying these criteria, a total of 1805 patients were included in the study, as illustrated in Figure 1.

**Figure 2:**
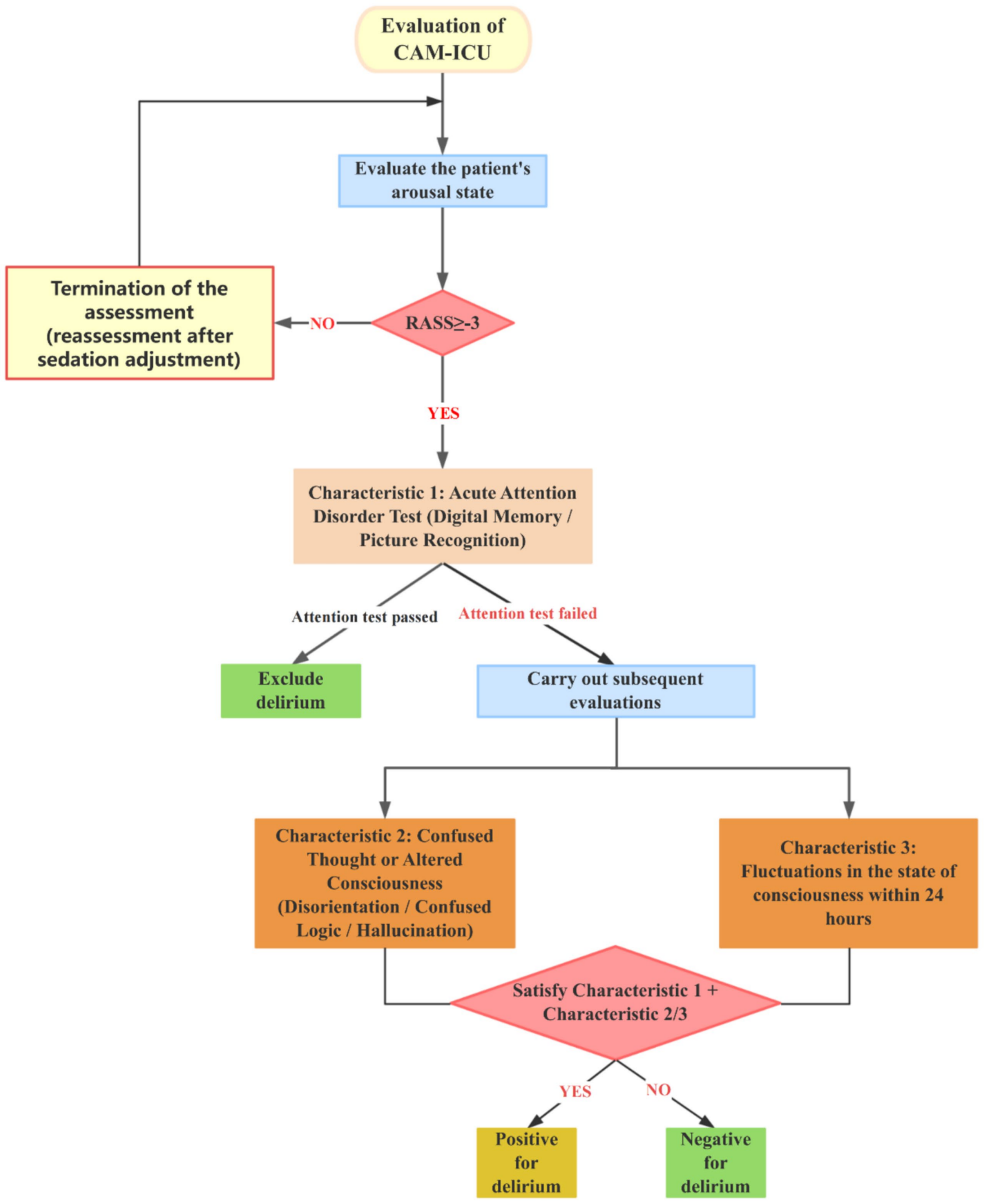
Flowchart of CAM-ICU delirium assessment.

The CAM-ICU (Confusion Assessment Method for the Intensive Care Unit) is a standardized bedside assessment tool specifically designed to rapidly identify delirium in ICU patients. The assessment process involves the following critical steps: (1) Evaluating acute onset of attentional impairment (which can be validated using tests such as digit recall or picture recognition); (2) Confirming disorganized thinking or changes in the level of consciousness; (3) Observing fluctuations in the course of the illness; (4) Excluding interference from sedation or coma on the assessment results (requiring a comprehensive evaluation based on sedation scores, such as RASS). Prior to initiating the assessment, it is crucial to ensure that the patient is arousable (RASS ≥ −3) (19). A positive delirium determination is made if the patient can cooperate in completing the attention test and simultaneously meets criteria 2 and 3 (20) (Figure 2).

### 2.3 Variable factors

Through a comprehensive review of the literature and clinical expertise, we identified and incorporated several potential confounding factors that may influence mortality outcomes as variables in our analysis of elderly delirium patients. These variables included demographic characteristics (gender, age), vital signs (heart rate, systolic blood pressure, diastolic blood pressure, mean arterial pressure, respiratory rate, oxygen saturation), laboratory parameters (blood glucose, hemoglobin, platelets, albumin, anion gap, bicarbonate, blood urea nitrogen, calcium, chloride, serum creatinine, sodium, potassium), patient comorbidity profiles (hypertension, myocardial infarction, congestive heart failure, peripheral vascular disease, cerebrovascular disease, chronic lung disease, rheumatism, peptic ulcer disease, mild liver disease, diabetes, kidney disease, malignant tumor, severe liver disease, metastatic solid tumor, AIDS), scoring systems within 24 h of ICU admission (Charlson Comorbidity Index, APSIII, SAPSII, OASIS), and post-admission treatment interventions (renal replacement therapy, mechanical ventilation, vasoactive drugs, benzodiazepine sedatives). Notably, the vital signs and laboratory parameters were based on the average values measured within the first 24 h after admission. Mechanical ventilation encompassed bilevel positive airway pressure (BiPAP) mask ventilation, endotracheal tube ventilation, T-piece ventilation, tracheostomy mask ventilation, and tracheostomy tube ventilation. Benzodiazepine sedatives specifically included diazepam, alprazolam, clonazepam, lorazepam, temazepam, midazolam, oxazepam, and chlordiazepoxide.

### 2.4 Calculation of LANR

The LANR was calculated using the formula: Lymphocyte × Albumin/Neutrophil (21). All admission-related values were extracted from the MIMIC IV database.

### 2.5 Statistical analysis

Participants were divided into three categories based on the tertiles of LANR: Group T1 (LANR≤0.21, *n* = 601), Group T2 (0.21 < LANR ≤ 0.47, *n* = 601), and Group T3 (LANR > 0.47, *n* = 602). Categorical variables were reported as proportions (%), with intergroup variations examined using chi-square or Fisher’s exact tests. Normally distributed continuous data were presented as mean ± standard deviation and compared using independent sample *t*-tests; non-normally distributed continuous data were expressed as median (Q1, Q3) and analyzed via the Wilcoxon rank sum test.

Covariates for the multivariate Cox regression model were selected based on univariate analysis results (Table 1) and clinical relevance. The final adjusted variables included age, gender, heart rate, SpO2, creatinine, peripheral vascular disease, diabetes, chronic pulmonary disease, myocardial infarct, congestive heart failure, benzodiazepines use, continuous renal replacement therapy (CRRT), and mechanical ventilation.

**Table 1:** Univariate analysis.

Variable	30-day mortality		365-day mortality	
	HR (95%CI)	*p*-value	HR (95%CI)	*p*-value
Age	1.02 (1.01,1.03)	<0.001	1.03 (1.02,1.03)	<0.001
Gender (male)	1.01 (0.85,1.20)	0.928	0.92 (0.8,1.05)	0.203
Heart rate	1.02 (1.01,1.02)	<0.001	1.01 (1.01,1.01)	<0.001
Systolic blood pressure	0.98 (0.97,0.99)	<0.001	0.98 (0.98,0.99)	<0.001
Diastolic blood pressure	0.99 (0.98,1)	0.011	0.99 (0.98,1)	<0.001
Average blood pressure	0.98 (0.97,0.99)	<0.001	0.98 (0.97,0.99)	<0.001
Respiratory rate	1.07 (1.05,1.09)	<0.001	1.06 (1.04,1.07)	<0.001
SpO2	0.9 (0.87,0.93)	<0.001	0.93 (0.91,0.96)	<0.001
Glucose	1.00 (1.00,1.00)	0.378	1.00 (1.00,1.00)	0.496
Hemoglobin	0.9 (0.87,0.94)	<0.001	0.89 (0.87,0.92)	<0.001
Platelets	1.00 (1.00,1.00)	0.136	1.00 (1.00,1.00)	0.33
WBC	1.01 (1.01,1.02)	<0.001	1.01 (1.01,1.02)	<0.001
Aniongap	1.06 (1.04,1.08)	<0.001	1.03 (1.02,1.05)	<0.001
Bicarbonate	0.98 (0.96,0.99)	0.009	0.99 (0.97,1)	0.06
BUN	1.01 (1.01,1.01)	<0.001	1.01(1.01,1.01)	<0.001
Calcium	0.98 (0.88,1.09)	0.692	0.99 (0.91,1.07)	0.758
Chloride	1.00 (0.99,1.01)	0.836	1.00(0.99,1.01)	0.429
Creatinine	1.12 (1.07,1.17)	<0.001	1.09 (1.06,1.13)	<0.001
Sodium	1.02 (1,1.03)	0.007	1.01 (1,1.02)	0.012
Potassium	1.3 (1.15,1.46)	<0.001	1.22 (1.12,1.34)	<0.001
Hypertension	0.99 (0.80,1.23)	0.943	0.88 (0.75,1.04)	0.142
Myocardial infarct	1.15 (0.94,1.4)	0.165	1.1 (0.94,1.27)	0.242
Congestive heart failure	1.3 (1.1,1.55)	0.003	1.42 (1.24,1.62)	<0.001
Peripheral vascular disease	1.02 (0.79,1.32)	0.882	1.08 (0.89,1.31)	0.429
Cerebrovascular disease	0.8 (0.64,1)	0.049	0.89 (0.76,1.05)	0.185
Chronic pulmonary disease	1.08 (0.89,1.3)	0.446	1.08 (0.94,1.25)	0.277
Rheumatic disease	1.83 (1.28,2.6)	<0.001	1.42 (1.05,1.92)	0.023
Peptic ulcer disease	1.11 (0.73,1.69)	0.621	1.16 (0.85,1.59)	0.348
Mild liver disease	1.74 (1.37,2.22)	<0.001	1.52 (1.25,1.85)	<0.001
Diabetes	0.99 (0.83,1.19)	0.932	1.08 (0.95,1.24)	0.252
Renal disease	1.24 (1.03,1.48)	0.021	1.31 (1.14,1.5)	<0.001
Malignant cancer	1.82 (1.49,2.23)	<0.001	1.9 (1.62,2.22)	<0.001
Severe liver disease	2.68 (1.95,3.67)	<0.001	2.43 (1.86,3.18)	<0.001
Metastatic solid tumor	2.16 (1.68,2.78)	<0.001	2.09 (1.7,2.58)	<0.001
Charlson Comorbidity Index	1.14 (1.11,1.18)	<0.001	1.15 (1.12,1.18)	<0.001
APSIII	1.02 (1.02,1.03)	<0.001	1.02 (1.02,1.02)	<0.001
SAPSII	1.04 (1.03,1.05)	<0.001	1.03 (1.03,1.04)	<0.001
OASIS	1.04 (1.03,1.05)	<0.001	1.03 (1.02,1.03)	<0.001
CRRT	1.65 (1.12,2.43)	0.011	1.3 (0.93,1.82)	0.119
Mechanical ventilation	0.74 (0.57,0.96)	0.024	1.08 (0.91,1.29)	0.369
Vasoactive drugs	1.54 (1.29,1.83)	<0.001	1.33 (1.17,1.52)	<0.001
Benzodiazepines	1.68 (1.4,2.01)	<0.001	1.32 (1.15,1.51)	<0.001
Length of Hospital stay	0.95 (0.94,0.96)	<0.001	0.99(0.99,1.00)	0.019
Length of ICU stay	0.99 (0.98,1)	0.13	1.00(0.99,1.01)	0.61

A multivariate Cox proportional hazards model was utilized to assess the impact of LANR on 30-day and 365-day mortality. Survival differences were visualized using Kaplan–Meier curves, and restricted cubic spline (RCS) analysis was employed to explore the dose–response relationship between LANR and mortality. All analyses were conducted using R 4.1.2 and Free Statistics 2.1, with a *p*-value <0.05 considered statistically significant.

## 3 Results

### 3.1 Baseline characteristics

Table 2 provides a comprehensive overview and summary of the information regarding the included and excluded patients. A total of 1,804 elderly delirium patients were included in this study, of whom 995 were female, accounting for 55.2% of the total cohort. The average age of the overall cohort was 77.6 ± 7.9 years, with an ICU stay duration of 3.3 days [IQR 1.8–6.7 days]. Elderly delirium patients with different levels of LANR exhibited distinct clinical characteristics. Compared to patients with high LANR levels, those with low LANR levels had longer overall and ICU hospital stays and presented with more severe illness. Furthermore, delirium patients with low LANR levels had a higher risk of comorbidities, including chronic pulmonary diseases, mild liver disease, and metastatic solid tumors. In terms of vital signs, patients with low LANR levels had higher heart rates and respiratory rates. Laboratory tests showed that low LANR patients had elevated white blood cell counts, suggesting the possibility of an inflammatory response. Additionally, these patients had higher levels of blood urea nitrogen and creatinine, indicating potential renal dysfunction.

**Table 2:** Baseline characteristics of patients.

Variables	Total *n* = 1804	T1 (LANR ≤ 0.21) *n* = 601	T2 (0.21 < LANR ≤ 0.47) *n* = 601	T3 (LANR > 0.47) *n* = 602	*P*-value
Demographics					
Age	77.6 ± 7.9	77.3 ± 8.0	78.0 ± 8.0	77.5 ± 7.8	0.366
Gender, *n* (%)					0.542
Female	995 (55.2)	327 (54.4)	325 (54.1)	343 (57)	
Male	809 (44.8)	274 (45.6)	276 (45.9)	259 (43)	
Vital signs					
Heart rate (beats/min)	85.3 ± 16.9	88.6 ± 17.7	84.9 ± 16.7	82.3 ± 15.7	**<0.001**
Systolic blood pressure (mmHg)	118.8 ± 17.0	114.4 ± 15.3	119.6 ± 17.1	122.3 ± 17.5	**<0.001**
Diastolic blood pressure (mmHg)	62.6 ± 11.0	61.3 ± 10.5	62.3 ± 11.1	64.1 ± 11.2	**<0.001**
Average blood pressure (mmHg)	78.1 ± 10.8	76.0 ± 9.9	78.1 ± 10.7	80.2 ± 11.2	**<0.001**
Respiratory rate (breath/min)	20.3 ± 3.9	20.9 ± 4.1	20.3 ± 4.0	19.7 ± 3.6	**<0.001**
SpO2 (%)	96.8 ± 2.2	96.6 ± 2.2	96.8 ± 2.3	96.9 ± 2.1	0.071
Laboratory indicators					
Glucose (mg/dL)	148.4 ± 54.5	151.3 ± 56.2	148.8 ± 53.6	145.2 ± 53.6	0.144
Hemoglobin (g/dL)	10.6 ± 2.2	10.4 ± 2.2	10.6 ± 2.0	10.9 ± 2.2	**<0.001**
Platelet (10^9^/L)	192.5 (137.0, 258.5)	195.0 (134.0, 277.0)	202.5 (148.0, 270.5)	180.5 (131.6, 234.0)	**<0.001**
WBC (10^9^/L)	11.7 (8.7, 15.9)	15.2 (11.4, 21.0)	11.6 (9.0, 15.0)	9.3 (6.7, 12.2)	**<0.001**
Anion gap (mEq/L)	15.5 ± 4.3	16.3 ± 4.7	15.4 ± 4.2	14.9 ± 3.9	**<0.001**
Bicarbonate (mmol/L)	21.9 ± 5.0	20.9 ± 5.3	22.1 ± 4.9	22.8 ± 4.6	**<0.001**
BUN (mg/dL)	28.5 (18.5, 47.5)	33.5 (22.5, 56.0)	28.5 (18.5, 46.5)	24.0 (16.0, 38.5)	**<0.001**
Calcium (mg/dL)	8.4 ± 0.8	8.2 ± 0.8	8.4 ± 0.8	8.6 ± 0.8	**<0.001**
Chloride (mEq/L)	103.7 ± 7.5	103.7 ± 7.3	103.7 ± 7.8	103.8 ± 7.6	0.905
Creatinine (mg/dL)	1.2 (0.9, 2.0)	1.4 (0.9, 2.4)	1.2 (0.8, 2.0)	1.1 (0.8, 1.6)	**<0.001**
Sodium (mEq/L)	139.1 ± 6.7	138.8 ± 6.5	139.0 ± 6.8	139.5 ± 6.7	0.132
Potassium (mEq/L)	4.3 ± 0.7	4.4 ± 0.7	4.3 ± 0.7	4.3 ± 0.7	**0.004**
Comorbidities, *n* (%)					
Hypertension, *n* (%)	387 (21.5)	118 (19.6)	136 (22.6)	133 (22.1)	0.403
Myocardial infarction, *n* (%)	429 (23.8)	149 (24.8)	160 (26.6)	120 (19.9)	**0.019**
Congestive heart failure, *n* (%)	728 (40.4)	259 (43.1)	248 (41.3)	221 (36.7)	0.067
Peripheral vascular disease, *n* (%)	234 (13.0)	82 (13.6)	81 (13.5)	71 (11.8)	0.572
Cerebrovascular disease, *n* (%)	373 (20.7)	89 (14.8)	132 (22)	152 (25.2)	**<0.001**
Chronic pulmonary disease, *n* (%)	496 (27.5)	193 (32.1)	138 (23)	165 (27.4)	**0.002**
Rheumatic disease, *n* (%)	76 (4.2)	32 (5.3)	26 (4.3)	18 (3)	0.129
Peptic ulcer disease, *n* (%)	73 (4.0)	30 (5)	21 (3.5)	22 (3.7)	0.351
Mild liver disease, *n* (%)	187 (10.4)	80 (13.3)	55 (9.2)	52 (8.6)	**0.014**
Diabetes, *n* (%)	657 (36.4)	214 (35.6)	218 (36.3)	225 (37.4)	0.813
Renal disease, *n* (%)	583 (32.3)	197 (32.8)	201 (33.4)	185 (30.7)	0.577
Malignant cancer, *n* (%)	290 (16.1)	122 (20.3)	68 (11.3)	100 (16.6)	**<0.001**
Severe liver disease, *n* (%)	73 (4.0)	32 (5.3)	18 (3)	23 (3.8)	0.115
Metastatic solid tumor, *n* (%)	137 (7.6)	59 (9.8)	38 (6.3)	40 (6.6)	**0.041**
Severity scores					
Charlson Comorbidity Index	7.5 ± 2.5	7.7 ± 2.6	7.4 ± 2.3	7.4 ± 2.6	**0.049**
APSIII	63.1 ± 25.1	69.9 ± 25.1	62.9 ± 24.7	56.5 ± 23.7	**<0.001**
SAPSII	46.8 ± 13.4	50.6 ± 13.6	46.0 ± 12.7	43.7 ± 13.0	**<0.001**
OASIS	38.3 ± 8.9	40.0 ± 8.8	38.5 ± 8.9	36.5 ± 8.7	**<0.001**
Treatment					
CRRT, *n* (%)	64 (3.5)	30 (5)	17 (2.8)	17 (2.8)	0.064
Mechanical ventilation, *n* (%)	278 (15.4)	101 (16.8)	85 (14.1)	92 (15.3)	0.439
Vasoactive drugs, *n* (%)	720 (39.9)	293 (48.8)	236 (39.3)	191 (31.7)	**<0.001**
Benzodiazepines, *n* (%)	972 (53.9)	333 (55.4)	325 (54.1)	314 (52.2)	0.524
Length of stay					
Length of Hospital stay (day)	11.6 (6.7, 20.2)	12.8 (7.6, 21.3)	10.6 (6.5, 20.4)	11.0 (6.1, 18.9)	**0.008**
Length of ICU stay (day)	3.3 (1.8, 6.7)	3.7 (1.9, 7.4)	3.2 (1.8, 6.8)	3.1 (1.6, 6.1)	**0.004**

SpO2, saturation of peripheral oxygen; WBC, white blood cell; BUN, blood urea nitrogen; APS III, acute physiology score III; SAPSII, simplified acute physiology score II; OASIS, Oxford acute severity of illness score. Values in bold indicate statistically significant differences (*p* < 0.05).

### 3.2 Single-variable analysis

As shown in Table 1, univariate regression analysis demonstrated the association between various variables and 30-day mortality. Age, heart rate, respiratory rate, anion gap, creatinine, sodium, potassium, congestive heart failure, rheumatic disease, mild liver disease, renal disease, malignant cancer, severe liver disease, metastatic solid tumor, Charlson Comorbidity Index, APSIII, SAPSII, OASIS, CRRT, vasoactive drugs, and benzodiazepines were positively associated with the 30-day mortality risk. In contrast, SpO2, hemoglobin, mechanical ventilation, and length of hospital stay were negatively associated with 30-day mortality risk.

Furthermore, the univariate analysis also revealed the association between various variables and 365-day mortality. Age, respiratory rate, anion gap, creatinine, sodium, potassium, congestive heart failure, rheumatic disease, mild liver disease, renal disease, malignant cancer, severe liver disease, metastatic solid tumor, Charlson Comorbidity Index, APSIII, SAPSII, OASIS, vasoactive drugs, and benzodiazepines were positively associated with the 365-day mortality risk. Conversely, SpO2 and hemoglobin were negatively associated with the 365-day mortality risk.

### 3.3 Restricted cubic spline analysis

**Figure 3:**
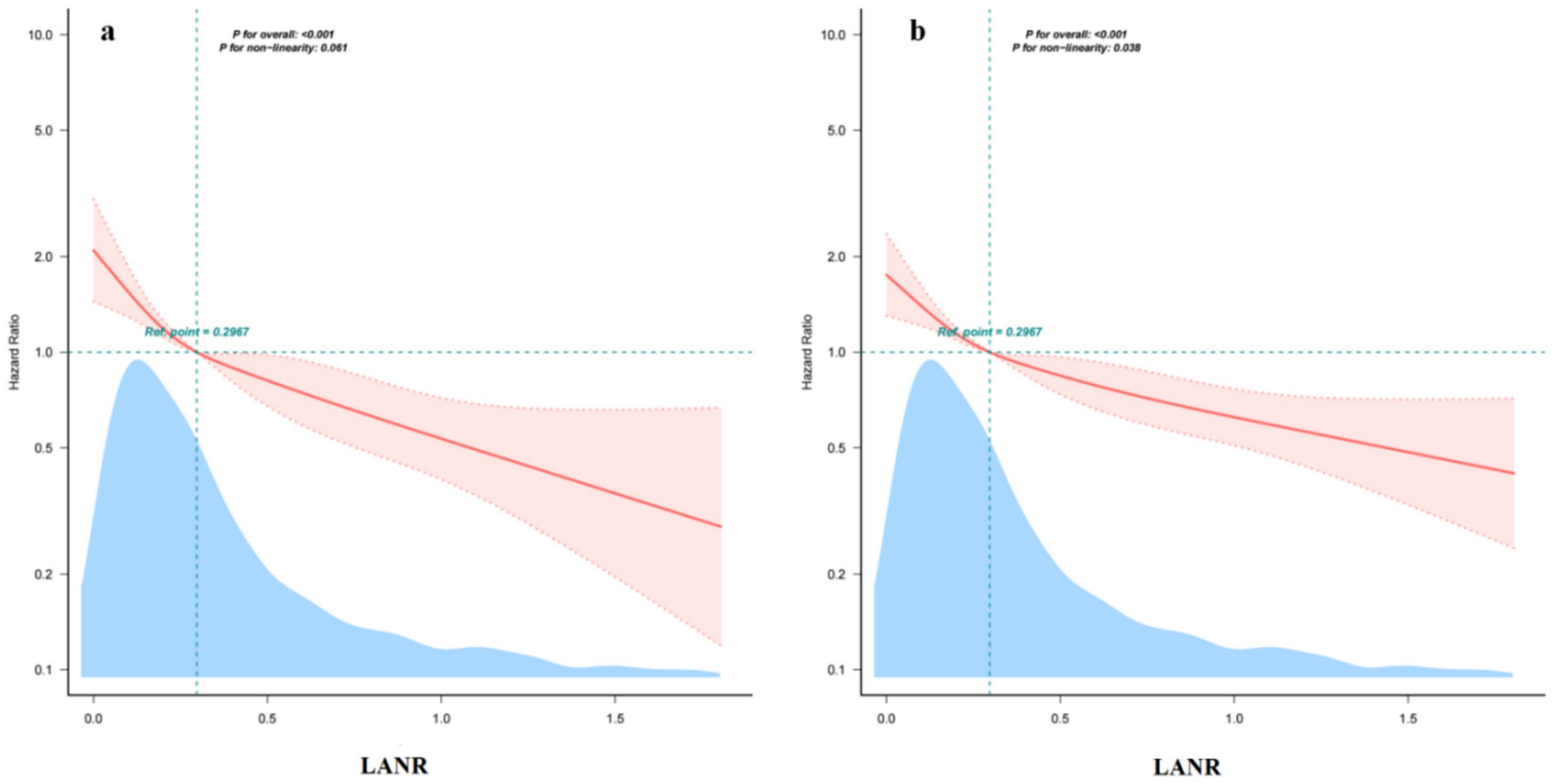
Fitted curves for the association between LANR and risk of death: **(a)** 30-day mortality and **(b)** 365-day mortality.

As shown in Figure 3, the potential association between LANR and both 30-day and 365-day mortality risks in elderly delirium patients was visually explored using restricted cubic spline analysis. The results indicated a linear relationship between LANR and reduced short-term mortality risk (*p* > 0.05). Although LANR showed a nonlinear relationship with long-term mortality risk (*p* < 0.05), the long-term mortality risk still decreased progressively as LANR levels increased.

### 3.4 Multivariable Cox regression analysis

As shown in Table 3, a Cox multivariable regression analysis was performed to assess the association between different levels of LANR and 30-day and 365-day mortality in elderly patients with delirium. After full adjustment for covariates, the study found that higher levels of LANR (T2, 0.21 < LANR ≤ 0.47) were associated with a 26% reduction in short-term mortality risk and an 18% reduction in long-term mortality risk [HR_30-day_ = 0.74, 95% CI: 0.60 ~ 0.91, *p* = 0.004; HR_365-day_ = 0.82, 95% CI: 0.70 ~ 0.96, *p* = 0.013]. Extremely high levels of LANR (T3, LANR > 0.47) were associated with a 45% reduction in short-term mortality risk and a 40% reduction in long-term mortality risk [HR_30-day_ = 0.55, 95% CI: 0.44 ~ 0.69, *p* < 0.001; HR_365-day_ = 0.60, 95% CI: 0.51 ~ 0.71, *p* < 0.001].

**Table 3:** Cox regression analysis.

Variables	Model 1		Model 2		Model 3	
	HR (95% CI)	*P*-value	HR (95% CI)	*P*-value	HR (95% CI)	*P*-value
30-day mortality						
T1 (LANR ≤ 0.21)	1 (Reference)		1 (Reference)		1 (Reference)	
T2 (0.21 < LANR ≤ 0.47)	0.7 (0.57 ~ 0.85)	<0.001	0.75 (0.61 ~ 0.92)	0.005	0.74 (0.6 ~ 0.91)	0.004
T3 (LANR > 0.47)	0.48 (0.38 ~ 0.6)	<0.001	0.55 (0.44 ~ 0.69)	<0.001	0.55 (0.44 ~ 0.69)	<0.001
*P* for trend		<0.001		<0.001		<0.001
365-day mortality						
T1 (LANR≤0.21)	1 (Reference)		1 (Reference)		1 (Reference)	
T2 (0.21 < LANR≤0.47)	0.78 (0.67 ~ 0.91)	0.001	0.82 (0.7 ~ 0.95)	0.011	0.82 (0.7 ~ 0.96)	0.013
T3 (LANR>0.47)	0.54 (0.46 ~ 0.64)	<0.001	0.6 (0.5 ~ 0.71)	<0.001	0.6 (0.51 ~ 0.71)	<0.001
*P* for trend		<0.001		<0.001		<0.001

Model 1: No Adjusted.

Model 2: Adjusted for Age, Gender, Heart rate, SpO2 and Creatinine.

Model 3: Adjusted for Model 2 Plus Peripheral vascular disease, Diabetes, Chronic pulmonary disease, Myocardial infarct, Congestive heart failure, Benzodiazepines, CRRT and mechanical ventilation.

### 3.5 Kaplan–Meier survival analysis

**Figure 4:**
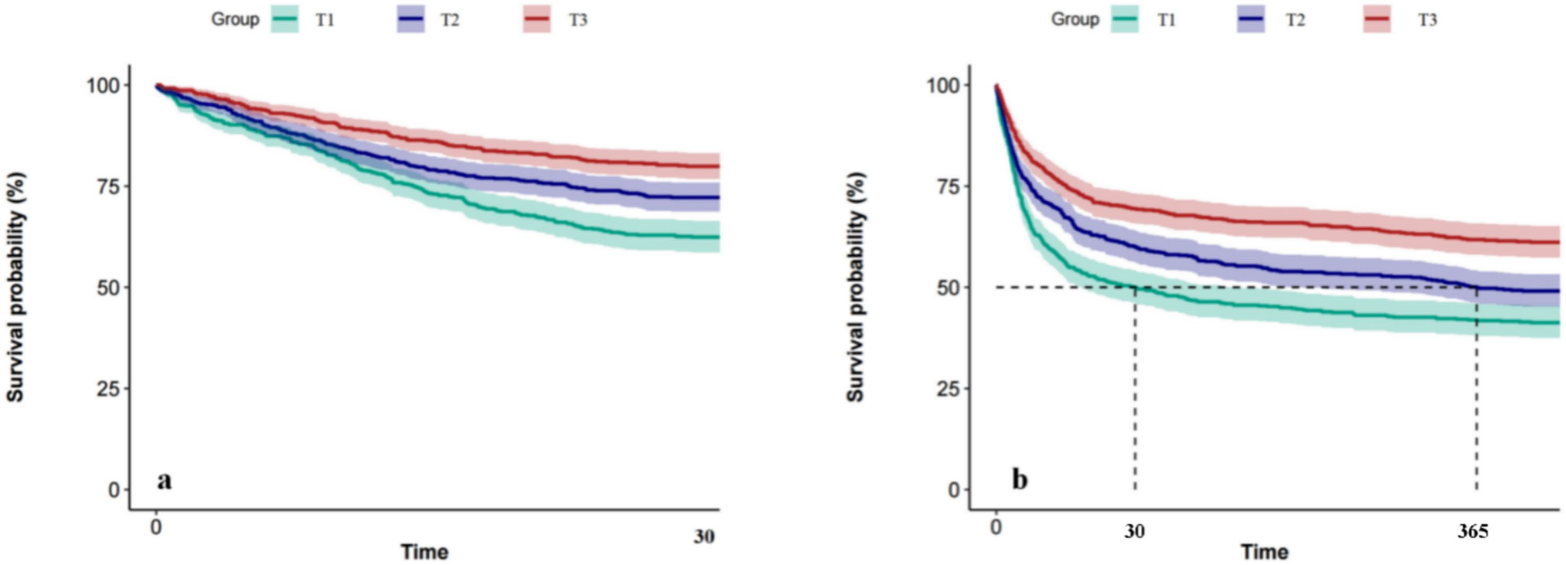
Kaplan–Meier survival analysis curves: **(a)** Prognosis within 30 days; **(b)** prognosis within 365 days.

As shown in Figure 4, Kaplan–Meier (KM) survival curves were plotted for elderly delirium patients 30 days (a) and 365 days (b) after ICU admission. The results showed that the survival probability on day 30 post-ICU admission decreased in the following order: T3 group, T2 group, and T1 group. Similarly, the survival probability on day 365 post-ICU admission followed the same order: T3 group, T2 group, and T1 group. These findings were consistent with the results of the Cox multivariate regression analysis.

### 3.6 Subgroup analysis

**Figure 5:**
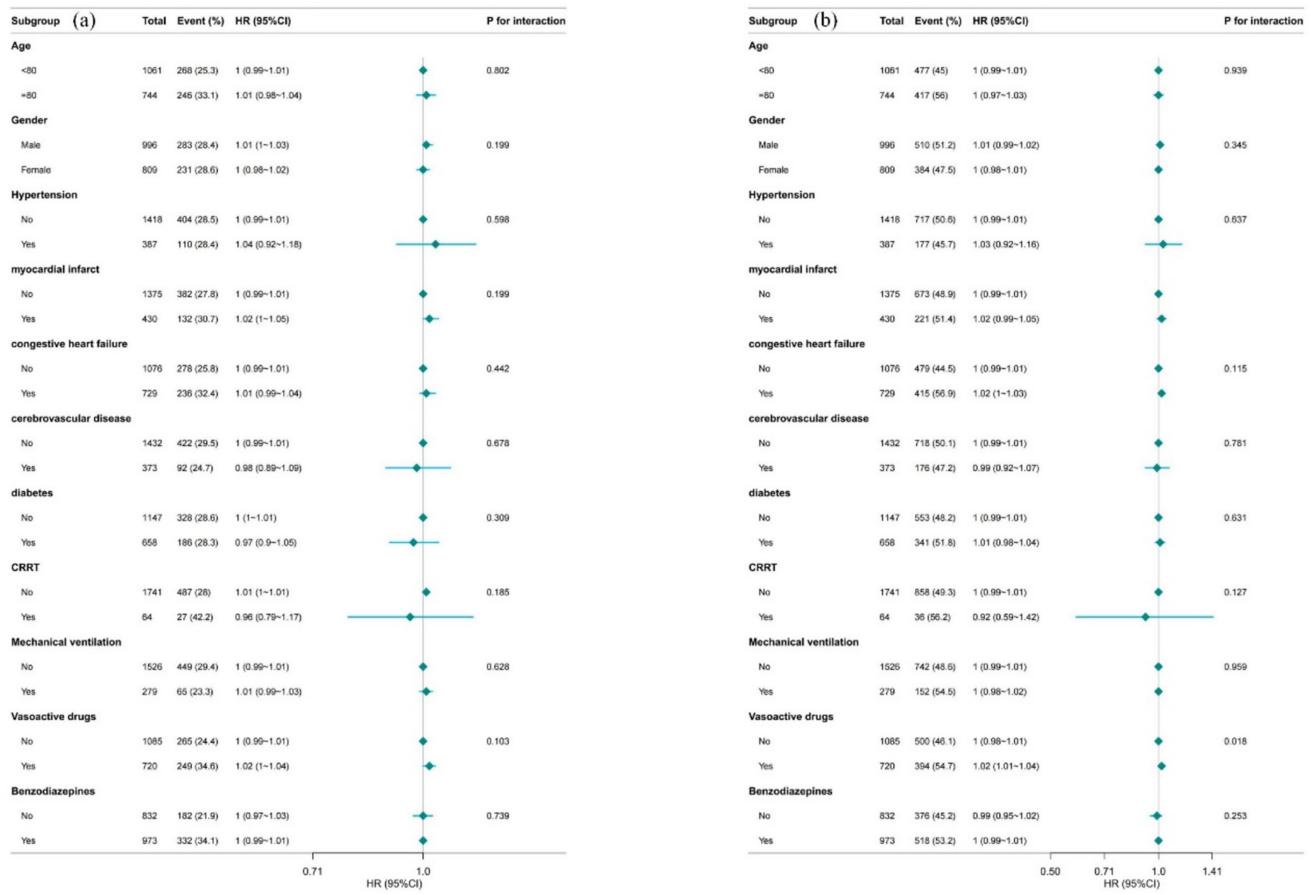
Forest plot **(a)** 30-day mortality; **(b)** 365-day mortality.

As shown in Figure 5, we performed Cox subgroup analyses with forest plots to evaluate the association between LANR and outcomes over different time horizons. The results demonstrated that while no significant interaction was observed between LANR and any confounder for 30-day mortality (all *p* > 0.05), a statistically significant interaction emerged specifically between LANR and vasoactive drug use for 365-day mortality (*p* = 0.018). These findings suggest that the interplay between vasoactive drugs and nutritional support may exert a more substantial influence on long-term prognosis—possibly through mechanisms involving chronic inflammatory regulation, organ recovery, and metabolic homeostasis—whereas short-term mortality remains predominantly driven by the acuity and severity of the critical illness itself.

### 3.7 ROC curve analysis

**Figure 6:**
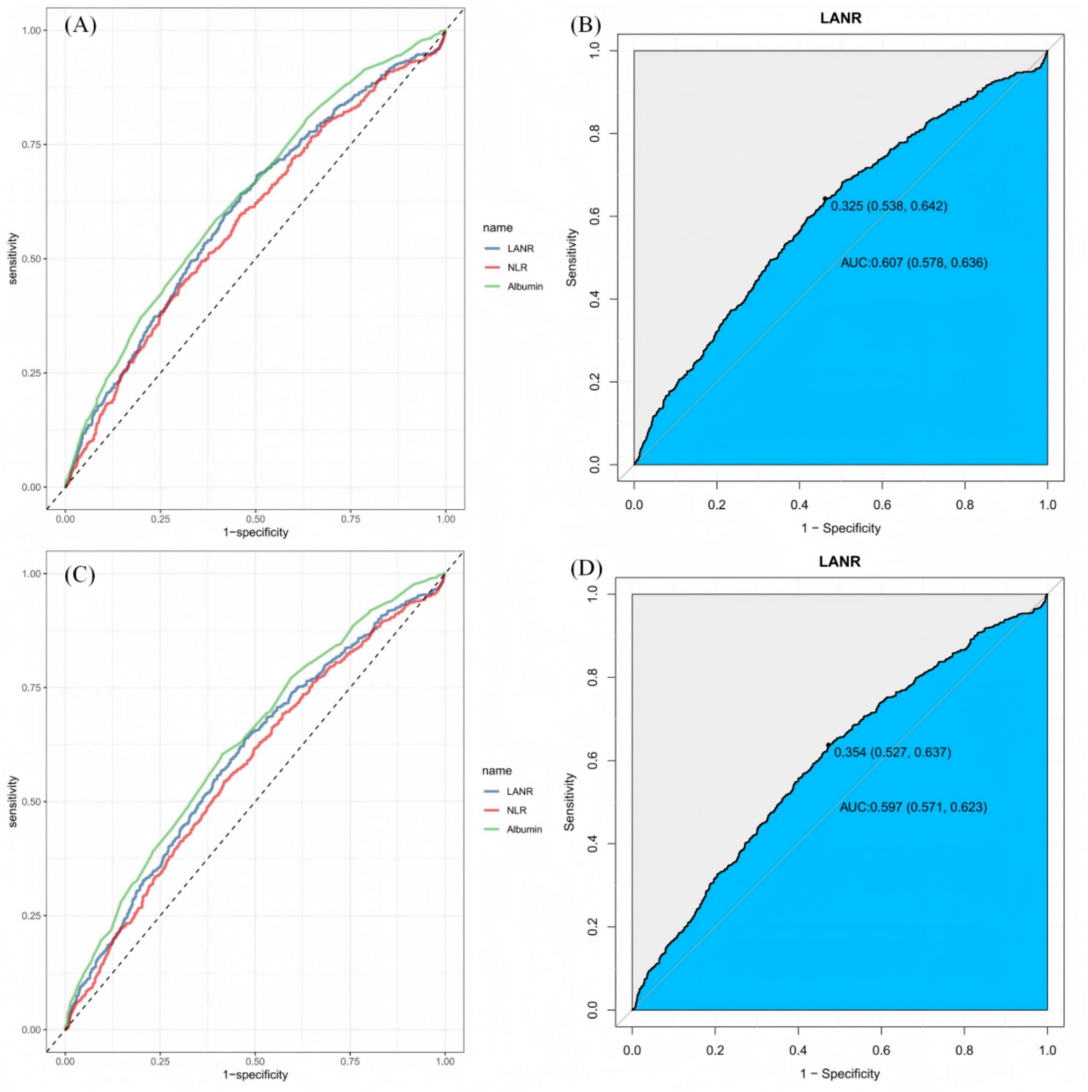
Receiver operating characteristic (ROC) curve analysis for mortality risk in elderly patients with delirium. **(A)** Comparison of the predictive performance of the lymphocyte-albumin-neutrophil ratio (LANR), neutrophil-to-lymphocyte ratio (NLR), and albumin for 30-day mortality risk. **(B)** Detailed ROC curve of LANR for predicting 30-day mortality risk. **(C)** Comparison of the predictive performance of LANR, NLR, and albumin for 365-day mortality risk. **(D)** Detailed ROC curve of LANR for predicting 365-day mortality risk.

This study evaluated the predictive efficacy of the LANR for mortality risk in elderly patients with delirium using ROC curve analysis. The results demonstrated that LANR achieved an AUC of 60.67% (95% CI: 57.75–63.58%) for 30-day mortality and 59.67% (95% CI: 57.06–62.29%) for 365-day mortality (Figure 6). While these values were comparable to the Neutrophil-to-Lymphocyte Ratio (NLR; 30-day: 58.50%; 365-day: 57.51%), they were slightly lower than those of albumin alone (30-day: 63.41%; 365-day: 62.70%). DeLong’s test indicated no statistically significant difference in AUC between LANR and albumin (*p* > 0.05). Although albumin exhibited marginally superior discriminative ability as a standalone marker, LANR integrates multidimensional information on inflammation, immunity, and nutrition. Its independent prognostic value, as confirmed in multivariate Cox regression analysis (see Table 3), underscores its clinical utility for risk stratification in this patient population.

## 4 Discussion

This study, leveraging data from the MIMIC-IV database, investigated the association between the lymphocyte-albumin-neutrophil ratio (LANR) and both short-term and long-term mortality in elderly patients with delirium. Our findings demonstrate a consistent inverse relationship between higher LANR levels and reduced mortality risk. Restricted cubic spline analysis revealed a linear inverse association between LANR and 30-day mortality risk; while a non-linear relationship was observed for 365-day mortality, the overall trend indicated a progressive decline in mortality risk with increasing LANR levels. Following adjustment for potential confounding variables, Cox proportional hazards models further confirmed that both moderately elevated and high LANR levels were significantly associated with lower 30-day and 365-day mortality rates. Furthermore, subgroup analyses identified a significant interaction effect between LANR and vasopressor use with respect to 365-day mortality. Collectively, these results suggest that LANR, as an inflammation-related biomarker, holds significant clinical prognostic value for predicting both short-term and long-term outcomes in elderly ICU patients with delirium.

The development of delirium is intricately linked to systemic and central nervous system inflammatory responses (22). Stressful events, such as surgery, infection, or trauma, can activate the peripheral immune system, leading to the release of pro-inflammatory cytokines (e.g., IL-6, TNF-α, and CRP). These cytokines transmit signals across the blood–brain barrier or via the vagus nerve, inducing microglial activation and central nervous system inflammation. This cascade ultimately results in neurotransmitter imbalance and synaptic dysfunction (23–25). Research indicates that in patients with delirium, the activity of Th2 and Treg cells within the immune regulatory system is compromised, potentially contributing to uncontrolled inflammatory responses (26). Elderly individuals frequently exhibit Alzheimer’s disease (AD)-related pathological changes, including β-amyloid protein deposition, tau protein phosphorylation, and vascular damage. These neurodegenerative alterations diminish the brain’s resilience to inflammatory or metabolic stress (“reduced brain reserve”) (27–29). Studies have demonstrated a significant association between preoperative elevations in neurodegenerative markers (e.g., neurofilament light chain protein, S100β) and the risk of postoperative delirium. Furthermore, abnormal cerebral blood flow may exacerbate neuronal injury (24, 30). Metabolic disturbances induced by surgery or infection can compromise the integrity of the blood–brain barrier, enabling peripheral inflammatory factors to infiltrate the central nervous system. This process is particularly prevalent in elderly patients with vascular aging, where oxidative stress and mitochondrial dysfunction may amplify delirium through the activation of inflammatory pathways, such as inflammasomes (31, 32). Additionally, the decline in cholinergic system function and excessive dopaminergic activity constitute the core pathological underpinnings of delirium. Inflammatory factors suppress acetylcholine release, while stress-induced increases in catecholamines further disrupt neurotransmitter balance, underscoring the critical role of inflammatory mediators in the onset and progression of delirium in older adults (33, 34).

Previous studies have consistently demonstrated that inflammation-based composite markers are closely associated with the occurrence and prognosis of delirium. For instance, the neutrophil-to-lymphocyte ratio (NLR), a well-established indicator of systemic inflammatory burden, has been shown to be positively correlated with delirium risk and adverse outcomes. In patients with acute ischemic stroke, an NLR > 5.6 was associated with a 2.3-fold increased risk of delirium, potentially mediated by neutrophil-driven neuroinflammation and disruption of the blood–brain barrier (35). Similarly, elevated NLR has been independently associated with short- and long-term mortality in delirium-related populations, supporting the role of excessive innate immune activation and lymphocyte suppression in delirium pathophysiology (35, 36).

Beyond inflammatory indices alone, nutritional status has also emerged as a critical determinant of delirium prognosis. Albumin, a key marker of nutritional reserve and anti-inflammatory capacity, has been shown to exert independent prognostic value. Previous studies reported that serum albumin levels below 3.5 g/dL were associated with a 1.8-fold increase in 30-day mortality among patients with delirium (37). Hypoalbuminemia may exacerbate oxidative stress, impair endothelial integrity, and increase blood–brain barrier permeability, thereby amplifying neuroinflammatory cascades (38). These findings are consistent with evidence demonstrating that nutrition-related indices, such as the geriatric nutritional risk index (GNRI), independently predict delirium risk and mortality in elderly ICU patients, although GNRI primarily reflects metabolic status without directly capturing inflammatory dynamics.

In this context, LANR represents an advancement over existing composite biomarkers by integrating inflammatory, immune, and nutritional dimensions into a single, comprehensive index. While traditional ratios such as NLR and the lymphocyte-to-monocyte ratio (LMR) mainly reflect immune–inflammatory balance, they do not account for metabolic or nutritional vulnerability. For example, Shi et al. reported that a higher LMR was associated with a reduced risk of delirium (OR = 0.82), yet their analysis did not consider the modulatory role of albumin on inflammation and physiological reserve (36). Likewise, studies by Kurniawan et al. (39) demonstrated that elevated NLR was associated with increased mortality; however, these studies were not restricted to delirium populations and did not control for nutritional status, potentially limiting their explanatory scope.

By incorporating albumin into the inflammatory–immune framework, LANR complements and expands upon these existing indices. An elevated LANR reflects a relative reduction in neutrophil-driven inflammation, preservation of adaptive immune capacity via higher lymphocyte counts, and adequate nutritional reserve indicated by higher albumin levels. This multidimensional integration is consistent with evidence showing that composite markers such as the C-reactive protein/albumin ratio (CAR) predict mortality, while suggesting that LANR may offer superior prognostic performance by capturing additional immune-related information (38, 40).

The inverse association between LANR and mortality observed in our study likely reflects synergistic effects across multiple biological pathways. A lower neutrophil burden may reduce the release of pro-inflammatory cytokines (e.g., IL-6 and TNF-α) and neutrophil-derived matrix metalloproteinase-9 (MMP-9), thereby limiting blood–brain barrier disruption and central inflammatory mediator diffusion (41, 42). Concurrently, higher lymphocyte levels—particularly regulatory T-cell subsets—may suppress excessive inflammation and promote immune homeostasis, a mechanism supported by studies linking elevated NLR to increased levels of S100B, a marker of blood–brain barrier injury (16, 35). Furthermore, albumin contributes to neuroprotection by scavenging reactive oxygen species, maintaining oncotic pressure, and reflecting hepatic synthetic function, all of which are essential for neurological resilience in critically ill older adults (43). In parallel, lymphocyte subsets such as CD4 + T cells are known to secrete brain-derived neurotrophic factor (BDNF), which supports synaptic plasticity and neural repair. These mechanisms collectively align with prior findings that higher LMR and lower NLR are associated with reduced delirium risk and improved outcomes (44, 45).

Collectively, our findings are consistent with existing evidence on inflammation- and nutrition-related composite biomarkers and extend the current understanding by integrating immune status into a unified prognostic framework. By consolidating three independently validated prognostic components—neutrophils, lymphocytes, and albumin—LANR provides a more comprehensive representation of the “inflammation–immunity–nutrition” axis underlying delirium vulnerability. This multidimensional nature may enhance its predictive accuracy and biological plausibility, offering a mechanistically grounded explanation for its association with reduced short- and long-term mortality in elderly patients with delirium.

As a composite biomarker, LANR integrates information on both inflammatory response (neutrophils and lymphocytes) and nutritional status (albumin). Its elevation may indicate the restoration of immune homeostasis and enhancement of metabolic reserve, demonstrating superiority over single indicators. By capturing the integrated effects of pathophysiological states, LANR enables the construction of a more precise multi-dimensional risk stratification model. When combined with clinical assessment tools such as CAM-ICU, it facilitates the rapid identification of high-risk patients in emergency departments/ICUs and optimizes resource allocation. Meanwhile, physicians can undertake targeted therapies in accordance with the ratio of LANR. For instance, low albumin levels can be addressed by albumin supplementation to enhance colloid osmotic pressure, regulate endothelial function, and exert antioxidant effects, thereby mitigating brain injury (46, 47). Additionally, neutrophil infiltration and lymphocyte depletion, which are central mechanisms of delirium-associated neuroinflammation, can potentially be modulated through targeted interventions such as granulocyte colony-stimulating factor or immune-nutrition therapy, restoring immune balance (48). This approach has been preliminarily validated in the context of albumin and derived neutrophil-to-lymphocyte ratio (Alb-dNLR score) in esophageal cancer prognosis, supporting the feasibility of individualized treatment guided by composite indicators (49).

The LANR is a composite biomarker that integrates inflammatory response, immune function, and nutritional status, thereby providing a holistic reflection of host physiological homeostasis. Currently, LANR is primarily utilized in the domain of oncology prognosis and is recognized for its robust stability and clinical reliability. For example, in colorectal cancer patients, preoperative LANR effectively predicts overall survival (OS) and progression-free survival (PFS), with corresponding areas under the receiver operating characteristic curve of 0.6276 and 0.5963. Notably, low LANR levels are significantly associated with adverse outcomes, with a HR ranging from 0.551 to 0.697 (21, 50). Similarly, LANR has been validated as an independent prognostic factor in pancreatic ductal adenocarcinoma and breast cancer, offering critical insights for clinical decision-making, including surgical indication evaluation and adjuvant therapy selection (51). In the context of elderly delirium management, existing research has predominantly focused on alternative inflammation-related indices, such as the NLR and LMR, which have demonstrated utility in predicting delirium risk and stratifying patient populations. As a marker of systemic inflammatory burden, NLR exhibits a significant positive correlation with delirium incidence in elderly hospitalized patients, facilitating early identification of high-risk individuals (13, 52). Conversely, LMR demonstrates independent predictive efficacy in patients with sepsis- or stroke-associated delirium; its inclusion in risk assessment models enhances discriminatory performance (as evidenced by increased AUC), enabling optimized risk stratification and guiding preventive interventions such as nutritional support and anti-inflammatory therapies (36). While direct evidence supporting LANR’s application in elderly delirium management remains limited, theoretical advantages over NLR and LMR—indices that reflect only single pathophysiological dimensions—are apparent. By integrating lymphocyte count (immune status), neutrophil count (inflammatory response), and serum albumin level (nutritional status), LANR enables multidimensional assessment of the inflammation-immune-nutrition axis. This characteristic confers greater pathophysiological relevance and potential prognostic value in elderly ICU patients, a population characterized by prevalent inflammation and nutritional imbalance. Ratio-based biomarkers derived from routine hematological parameters quantify immune-inflammatory dysregulation, providing clinicians with practical, actionable risk assessment tools. In clinical practice, these indices inform individualized management strategies, including intensified delirium screening for high-risk patients, implementation of multimodal preventive or pre-rehabilitation interventions, and optimization of pharmacotherapy (e.g., minimizing exposure to deliriogenic agents such as benzodiazepines). Such approaches aim to reduce delirium incidence and improve clinical outcomes (36). Against this backdrop, our study is the first to investigate LANR in the prognostic evaluation of elderly delirium patients, revealing a significant inverse association between LANR and both short-term and long-term mortality risk. These findings expand LANR’s potential clinical utility beyond oncology. While prospective evidence supporting LANR-guided clinical management is lacking, our results suggest that LANR—an accessible, cost-effective, and information-rich inflammation-related biomarker—may complement the current biomarker landscape centered on NLR and LMR, offering novel evidence-based insights for prognosis and treatment decision-making in elderly ICU delirium patients.

## 5 Limitations

Although this study has demonstrated a significant association between lymphocyte-to-albumin-to-neutrophil ratio (LANR) and mortality in elderly patients with delirium, several limitations should be acknowledged with caution. Firstly, the lymphocyte–albumin–neutrophil ratio (LANR) and other baseline variables were derived from average values obtained within the first 24 h of intensive care unit (ICU) admission. While this approach is appropriate for evaluating short-term outcomes, it may fail to fully capture the dynamic physiological changes occurring during subsequent stabilization or recovery phases—changes that are particularly critical for long-term prognostic indicators such as 1-year mortality. Acute inflammatory and nutritional states at ICU admission may diverge from trajectories observed later in the disease course, and reliance on early, single-point measurements could therefore compromise the representativeness of LANR for long-term prognostic assessment. That said, early LANR levels may still reflect initial disease severity and physiological reserve, factors that exert potential sustained effects on long-term clinical outcomes. Future studies incorporating serial measurements and dynamic monitoring of LANR would help to more precisely characterize its temporal evolution patterns and further improve the predictive performance of long-term risk stratification in elderly patients with delirium. What’s more, This study is constrained by the inherent limitations of its retrospective observational design, which draws on data from a single database. While extensive covariate adjustments were performed, residual and unmeasured confounding factors cannot be fully eliminated, and the generalizability of the study findings to other clinical contexts may be restricted.

## 6 Future perspectives

The multidimensional nature of LANR suggests promising translational potential beyond delirium prognosis. Future studies should prioritize multi-center validation in pathophysiologically aligned conditions such as sepsis and cardiovascular diseases, where inflammation-immunity-nutrition interactions are prominent. Developing standardized protocols for dynamic LANR monitoring could enable real-time risk assessment and therapy guidance. Mechanistic research linking LANR to specific cytokine profiles and immune cell subsets would strengthen its biological plausibility. Ultimately, integrating LANR with established biomarkers may yield composite models for precision medicine approaches in critical care.

## 7 Conclusion

Higher levels of the inflammatory-nutritional marker LANR are associated with significantly lower short- and long-term ICU mortality in elderly patients with delirium. This inverse relationship underscores the potential clinical utility of LANR as a readily available prognostic biomarker.

## Data Availability

The original contributions presented in the study are included in the article/supplementary material, further inquiries can be directed to the corresponding author.
